# Supplementary material for: A self-powered analog sensor-data-logging device based on Fowler-Nordheim dynamical systems
Source: Nat Commun. 2020 Oct 28;11:5446. doi: 10.1038/s41467-020-19292-w (PMC7595237; doi:10.1038/s41467-020-19292-w)
Supplement: Supplementary file 1 — Supplementary Information [file 41467_2020_19292_MOESM1_ESM.pdf]

Supplementary Information

Supplementary Note 1: Different leakage mechanisms for dynamical logging devices

Three different types of dynamical systems are simulated based on different leakage element $I(V_t)$ in Fig. 1b. All systems would respond to an external signal (a square pulse), and then resynchronize to their baseline response. This is illustrated in Fig. 1a for three different leakage elements. When the leakage elements is a resistor, the dynamics follow an exponential characteristic. However, an extremely large resistance would be required to sustain the effects of the input pulse (or transient response). As an example, for a system with $C = 1\,\mathrm{pF}$, $R = 1\,\mathrm{T}\Omega$, $V_0 = 3\,\mathrm{V}$, a 1-second-long, $100\,\mathrm{mV}$ input signal will elicit a response that can be observed for 5.5 seconds. This is illustrated in Fig. 1b. Note that when the leakage element is a constant current (reverse biased diode leakage), the input pulse does not elicit any change in the response. For the leakage element based on FN tunneling, which follows a $1/\log(t)$ dynamics, the input pulse elicits a response that shows a much longer resynchronization time, as shown in Figs. 1a–b. This feature has been modeled and experimentally verified in the main text.

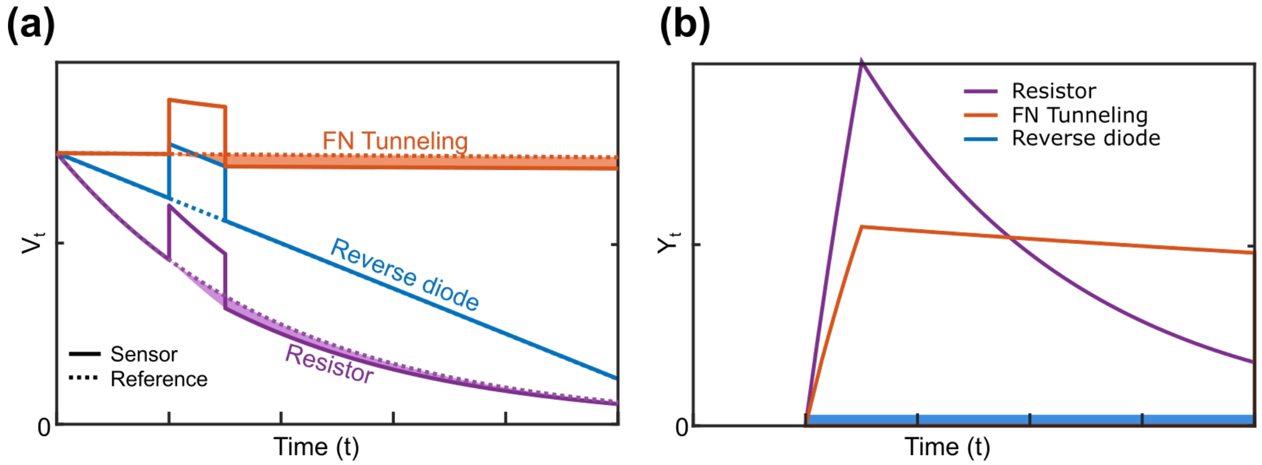

Supplementary Figure 1. a) Different leakage elements (Fowler-Nordheim tunneling, reverse biased diode and resistor) elicit different resynchronization responses. b) Note that the desynchronization response for the reverse diode case is zero.

Supplementary Note 2: Programming and synchronization

The differential sensor-data-logging system consists of two nodes: sensor and reference node. Each node contains two floating gates decoupled via a capacitor. The charge on the four gates of the system can be individually programmed using a combination of tunneling (increases charge, course) and hot electron injection (decreases charge, fine). The programming block for each gate is selected via a switch. Injection is initiated by setting V_{DD} = $7\,\mathrm{V}$, and setting the input pin to a value (via a DAC), such that V_{DS} is above $4.2\,\mathrm{V}$. V_{DS} can be modulated via the gate voltage because the PMOS is in a source follower configuration. Tunneling is realized by bring V_{tun} to a high potential. For programming the tunneling node to be in the FN tunneling regime, we used $V_{\mathrm{tun}} = 21\,\mathrm{V}$. Except for the self-powered experiments using piezo crystals, we did not have to program the tunneling node in FN tunneling regime using V_{prog} pin. Instead, we could set the input pin to a stable voltage (analog ground) which would push the node into FN regime. The DAC voltage was calculated for each run such that a tunneling node's potential at the start of each experiment was the same (as measured by the readout node). When the needed DAC voltage exceeded $5\,\mathrm{V}$, we would initiate tunneling. This process allowed us to limit the number of high voltage tunneling cycles and increase the experimental life of the recorder. This process cannot be done in actual deployment because there would not be an external DC source. Hence, for self-powered piezo experiments, we carried out tunneling for each trial.

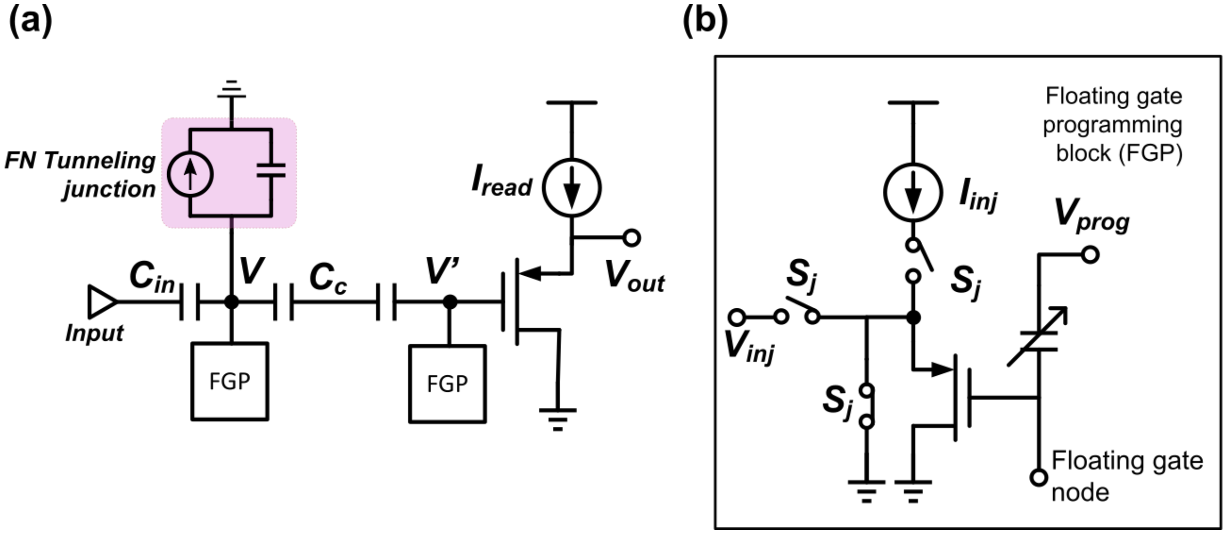

Supplementary Figure 2. Programming and synchronization: a) Each floating gate node can be individually programmed through the Floating Gate Programming (FGP) block. b) Electrons can be tunneled out of the floating gate by setting the V_{prog} to a high potential. Electrons can be injected into the floating gate via hot electron injection. The switch S_j, set via a shift register, allows for individual control of the injection channel. V_{inj} node is monitored during injection.

Supplementary Note 3: Device parameters and drift correction

Device parameters from equation 1 (Main text) can be experimentally obtained by allowing the floating gate to discharge via Fowler-Nordheim tunneling and fitting the model on observed data.

We obtained the following parameters:

Device No.	Node	$\log(k_1)$	k_2	k_3
1	Sensor	38.59	347.20	-4.24
	Ref	39.47	359.04	-4.43
2	Sensor	44.53	425.01	-4.86
	Ref	42.06	389.18	-4.57
3	Sensor	42.14	381.26	-4.35
	Ref	41.16	370.20	-4.30

Table I
DEVICE PARAMETERS

k_0 depends on the initial conditions. The starting voltages for each node can be chosen such that the sensor and reference have the same rates and are thus synchronized. However, the mismatch in other parameters causes the two nodes to drift. The drift is predictable and can be corrected as shown in Fig. 3. k_3 was subsumed into V_t by setting $V_t \rightarrow V_t - k_3$. The modified V_t is used for derivation of the explicit model in equation 4.

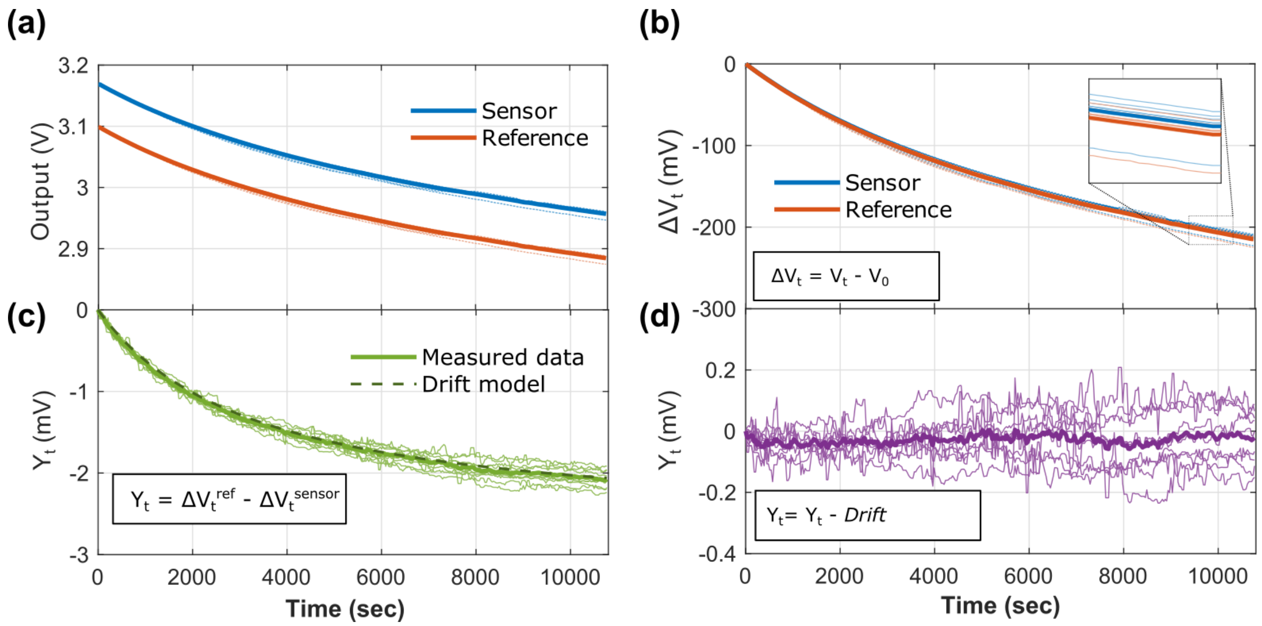

Supplementary Figure 3. Synchronization after correcting for drift: a) Experimentally measured values of sensor and reference output voltages. b) Change in sensor and reference values from the baseline $\Delta V_t = V_t - V_0$. c) Desynchronization between the sensing and reference nodes. A consistent drift is observed across trials which can be compensated. d) Desynchronization compensated for drift, which is the final recorder response.

Supplementary Note 4: Temperature compensation

To ensure accurate temperature compensation, it is important to accurately initialize the reference and the sensor devices. To illustrate this we show raw measured data from our experiments (Fig.2) for the sensor and the reference devices. The dataset shows an outlier due to incorrect initialization of the reference device (as highlighted in Fig. 4, where the reference node was incorrectly initialized lower than the target $50\,\mathrm{mV}$ difference from the sensor node). This difference results in improper compensation of the temperature variations. To ensure consistency, we have removed the outlier from Fig.2e.

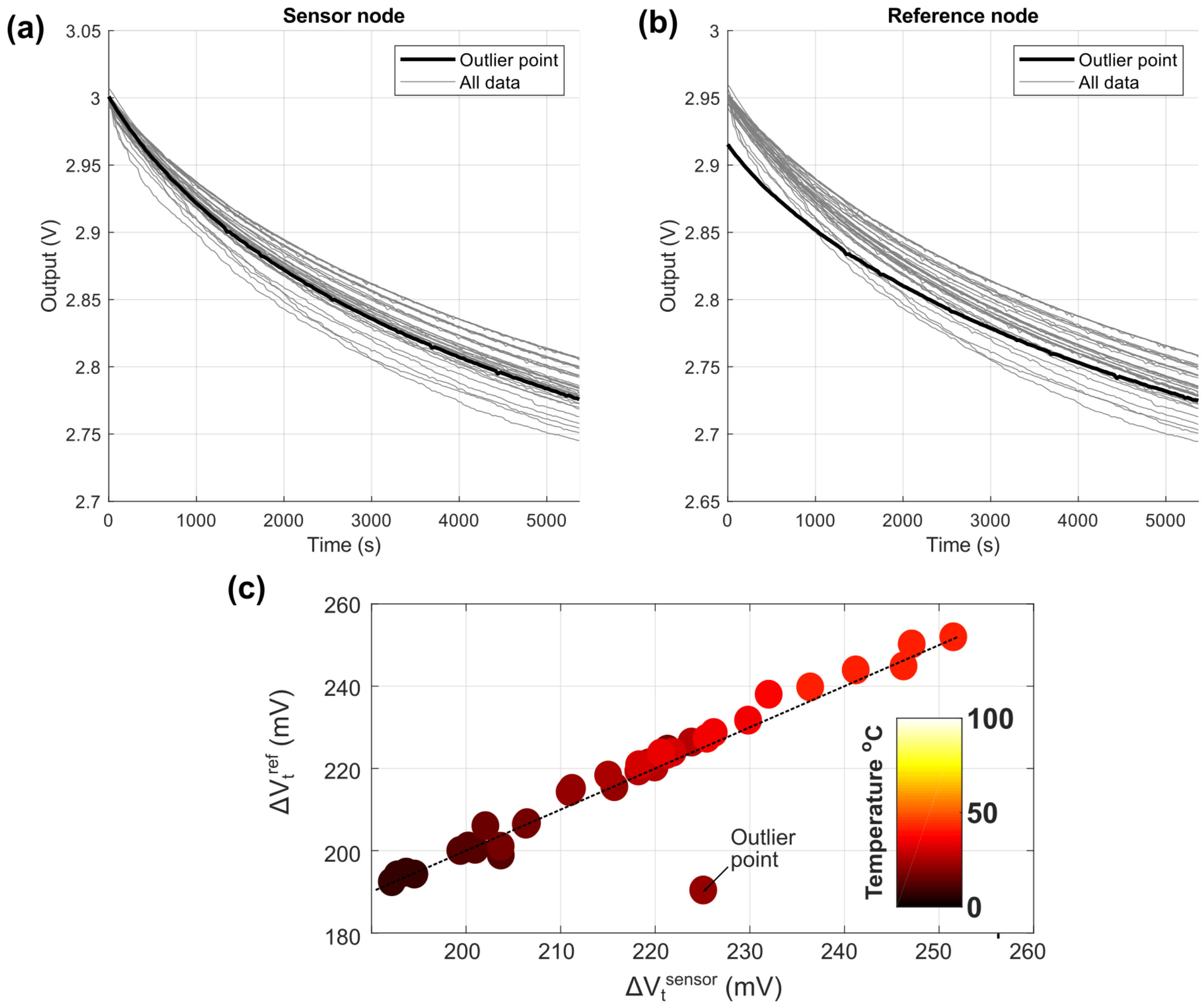

Supplementary Figure 4. a-b) Raw sensor and reference data for temperature compensation experiments highlighting the outlier which results in incorrect temperature compensation, as shown in c)

Supplementary Note 5: Model validation

The assumptions made in the derivation have been validated against a general ODE solver (Figs. 5 and 6). As shown in the figure, the error was less than $10\,\mu V$ for a response of $1.5\,mV$. Same analysis was run 100 times and the relative error was always less than $1\,\%$. The action model was computationally faster to solve than the ODE solver by a factor of 10^5. The explicit action model led to large errors when the input signal was large (Fig. 6). The error arises due to assumption $Y \ll V$, made during linearizing the equation 16, to estimate the resynchronization of the response. For large Y, higher order terms can no longer be ignored and the resynchronization will be faster. As the 1st order model ignores these terms, it always overestimates the expected action at time T. The error in the model can be empirically reduced by fitting a model between the expected response (as generated by the ODE solver) and response calculated by the action model (Fig. 6c).

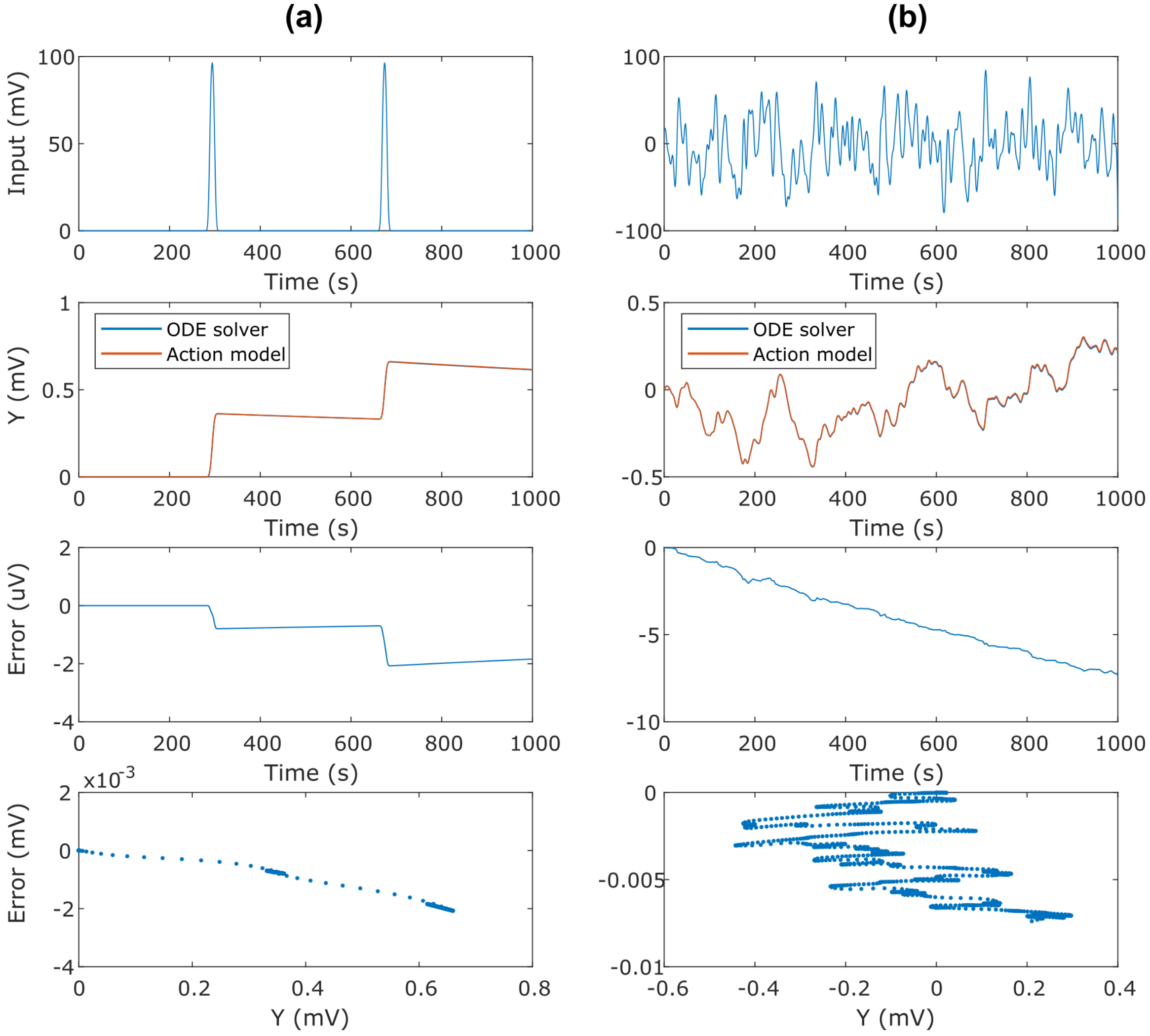

Supplementary Figure 5. Comparison between ODE solver and the action model for a) Short pulses and b) Small magnitude random signal. In either case, the action model closely tracks the ODE solver. The maximum error was less than $10\,\mu V$ for both cases.

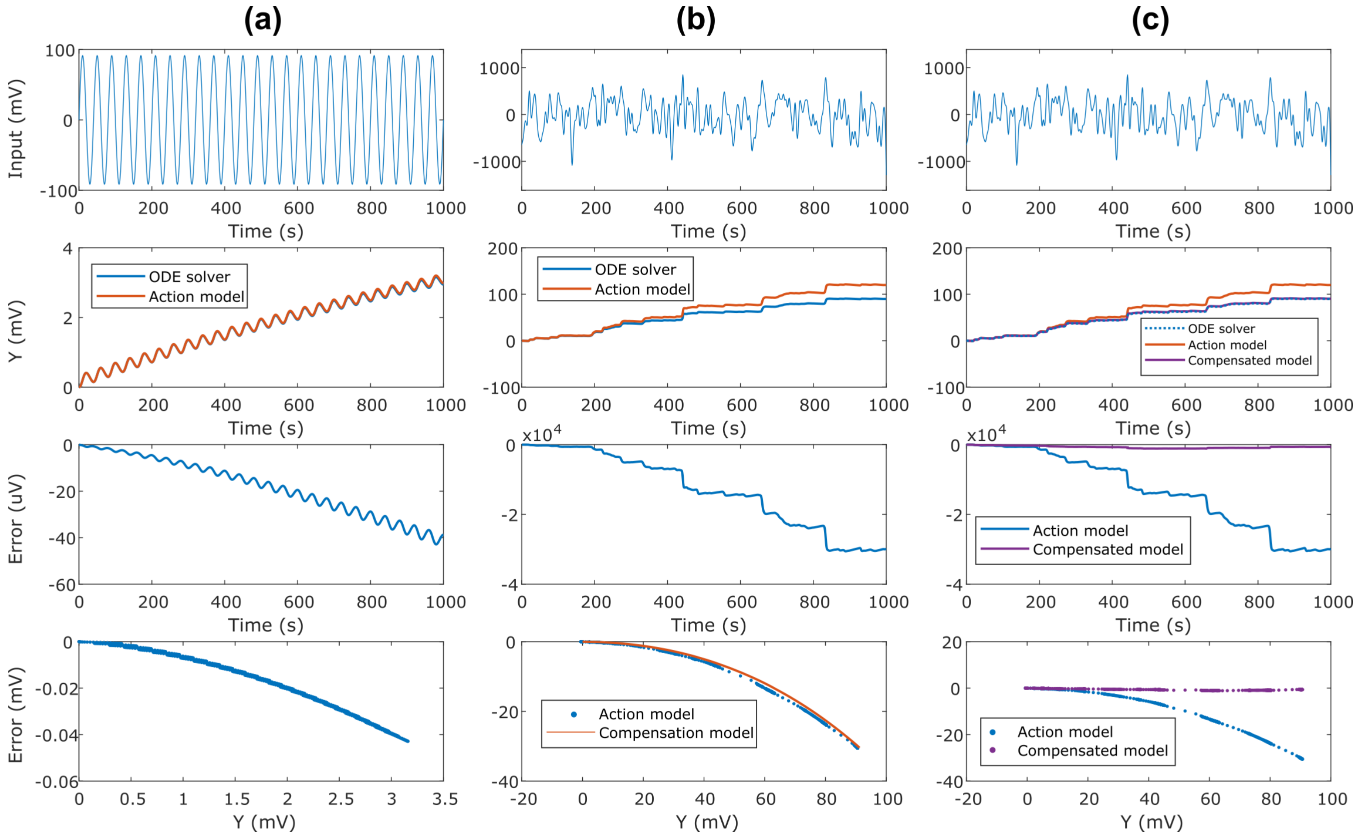

Supplementary Figure 6. Comparison between ODE solver and the action model a) Harmonic input signal leads to continuous increase in action, the error increases as action increases. b) Large continuous random signals lead to large errors in the action model compared to the ODE solver. However there is a relationship between the action and the error. An empirical model was fit that modeled error as a function of action. c) Compensation with this model leads to higher accuracy.

Supplementary Note 6: AC analysis

The action induced by an AC coupled signal is monotonic with the energy of the signal. Actions due to different waveform shapes are more similar for signals with same energy (Fig. 7b) compared to signals with same amplitude (Fig. 7a).

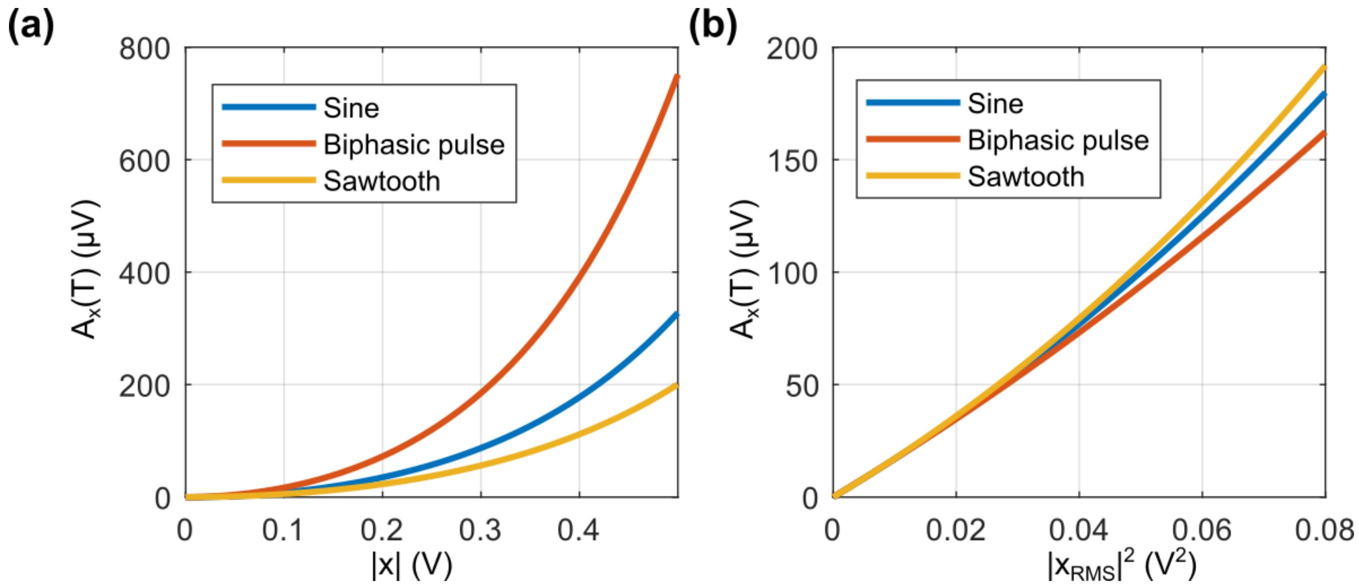

Supplementary Figure 7. Simulation results for recorder response to AC signals. a) Action induced by signals of different shapes as a function of amplitude of the signal. System is sensitive to biphasic signals because of the rectification induced by FN tunneling. b) Action induced by signals of different shapes as a function of energy of the signal.

Supplementary Note 7: Data retention model

Retention time for a given input signal was found by a fixed point method. First, a noise model was generated using experiments without any input modulation. Standard deviation (σ_{t}) was calculated across all runs as a function of time. Ideally, if the dynamics were perfectly synchronized, then the σ_{t} obtained would be 0. However, we find that σ_{t} increases with time due to integration of noise. We fit a rational equation on this noise.

$$\sigma_{\mathrm{t}} = \frac{at}{t+b}$$

We chose this equation so that it stays bounded as time approaches ∞. Total noise in the system is given by

$$N_{\mathrm{t}} = \sigma_{\mathrm{t}} + N_0$$

where N_0 is the noise associated with readout circuits and data acquisition system.

The time of retention T_{ret} was defined as the time instance at which the expected recorder response (Y_{t} becomes lower than the predicted noise in the system N_{t}, i.e. the signal-to-noise ratio goes below unity. Thus, at time $t = T_{\mathrm{ret}}$

$$Y_{T_{\mathrm{ret}}} = N_{T_{\mathrm{ret}}}$$

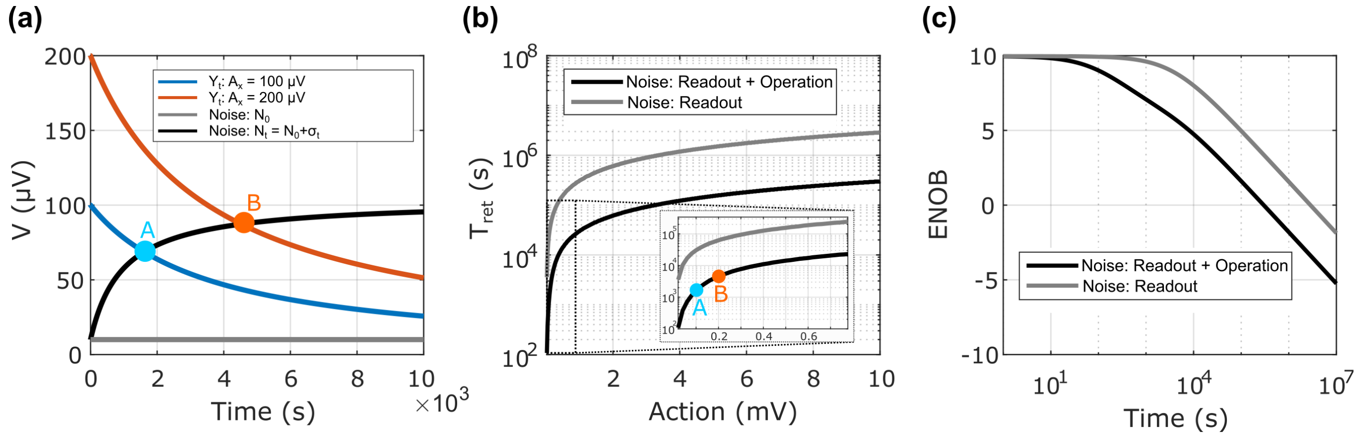

Supplementary Figure 8. a) Colored traces indicate recorder response for input signals with 100 and 200 μV actions. The gray trace indicates constant noise (N_0), due to readout and measurement circuits. Black curve indicates noise (N_{t}) due to unintended desynchronization occurring in absence of an input signal. It is obtained by adding the readout noise to the input referred noise (σ_{t}, assumed to be the standard deviation across trials for a recorder with no input signal - see variance in Fig. 2d). Intersection of the response curve with the noise curve (eg. Points A and B) is an estimation of system retention time, T_{ret}, at which point, the SNR of the system goes below 1. b) Retention time, T_{ret}, plotted as function of action for different noise profiles. Points A and B correspond to the intersection points in panel (a). T_{ret} varies exponentially with action. c) Amount of information, measured as the effective number of bits (ENOB), stored in the system which decreases with time.

Supplementary Note 8: Parametric analysis

Using modeling and simulations, we conducted parametric analysis for our system. Parameters T (Fig. 9a) and V_0 ((Fig. 9b) are operational parameters that can be set at run time according to application requirements. k_1 (Fig. 9c) depends on the area of the tunneling junction and on the capacitance associated with the floating gate node. k_1 and k_1 (Fig. 9d) are also influenced by the thickness of the insulating material and other material properties like the barrier height at the interface between the conductor and the insulator.

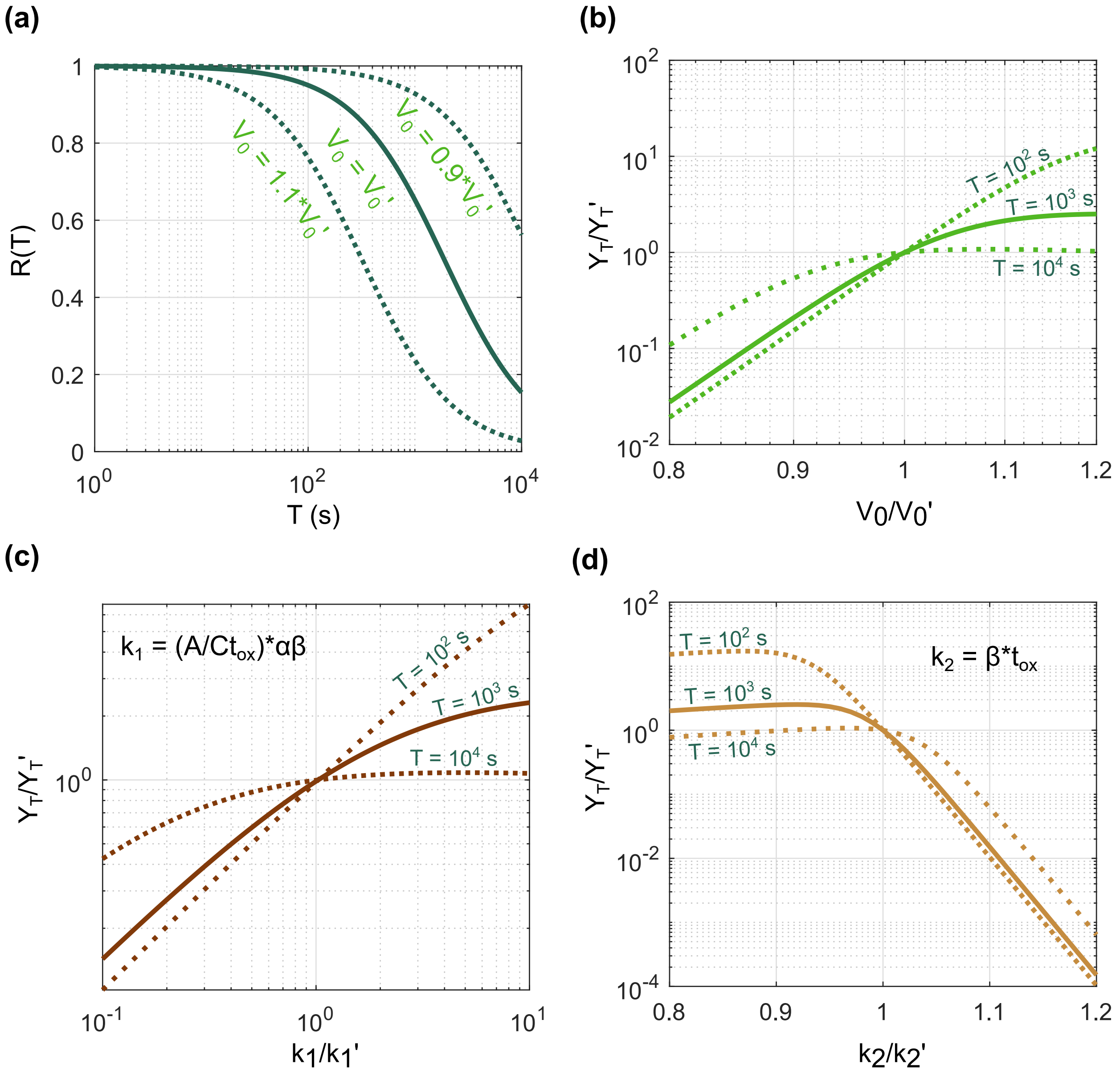

Supplementary Figure 9. Simulation results for sensitivity and parametric analysis. Default parameters are $k_1 = \exp(38.5), k_2 = 346, V_0 = 7.5V$. Y_T' is the baseline response at time T for a single square pulse of magnitude $100\,\mathrm{mV}$ and duration 1 s. a) Time of sampling determines the amount of resynchronization b) Initial programming voltage affects the sensitivity of the recorder, but its effect becomes attenuated as time of sampling increases (due to resynchronization) c,d) Device parameters k_1 and k_2 can be tuned via system design and material selection to optimize the recorder response. α and β are material parameters [1].

Supplementary Note 9: Impedance analysis and power estimation

We performed simulation studies to characterize the input impedance of our sensor logger which can be modeled by the equivalent circuit shown in Fig. 10a. The DC input impedance of our system is on the order of $10^{18}\,\Omega$, since the input is connected to the gate of a MOSFET and the FN tunneling current is in the order of attoamperes. In this case, the impedance of the ESD protection diodes dominate the input impedance at DC frequency. We ignore this leakage path for our analysis and the high pass cut-off frequency is found to be at $10^{-5}\,\mathrm{Hz}$ (Fig. 10b). At higher frequencies, the input capacitance and gate-to-substrate capacitance, along with input parasitic resistances create a low impedance path. However, this power is predominantly reactive in nature (Fig. 10c) and can be minimized with suitable source impedance matching. The power dissipated by the sensor-data-logger can be estimated as

$$P(\omega) = \Re\left(\frac{V^2}{2Z_{in}(\omega)}\right)$$

Assuming that the natural dynamics of the FN device lie less than the frequency range of 1mHz, the average power dissipated can be estimated over the signal bandwidth of $1\,\mathrm{mHz}$ to $1\,\mathrm{kHz}$ to be $0.05\,\mathrm{aW}$. So for an event lasting 100 seconds, energy dissipated is on the order of $5\,\mathrm{aJ}$.

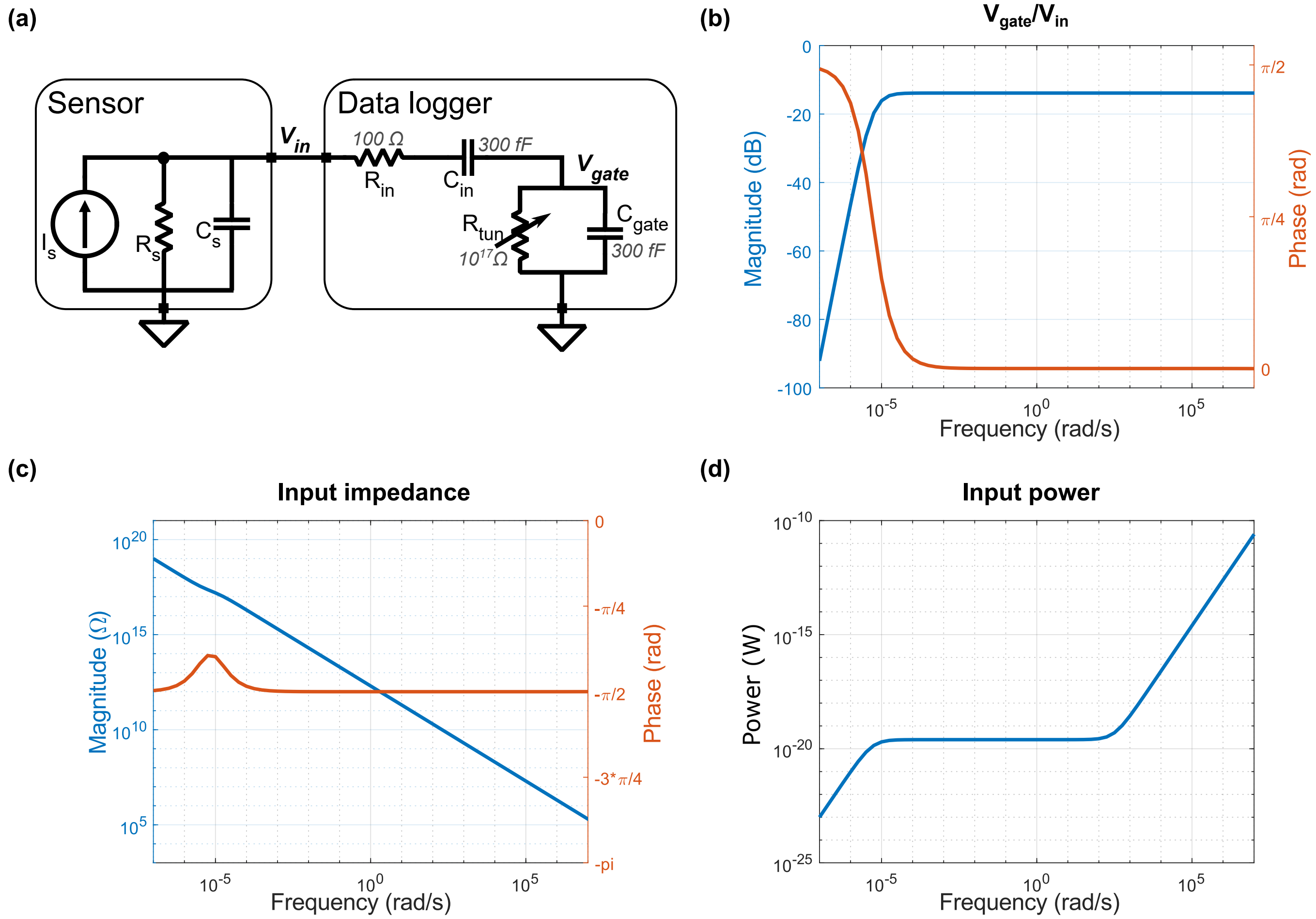

Supplementary Figure 10. a) Equivalent circuit model of a sensor interfaced with sensor-data-logger. b) Relation of V_{gate} to system input. c) Equivalent input impedance. d) Power spectral density for a matched system.

Supplementary Note 10: Temporal dependence

Fig. 11 shows the weak dependence of the recorder output to the time of occurrence of events. Earlier events lead to larger desynchronization, but have more time to recover. Modeling studies show that the net result is that later events lead to a larger output at readout. However, the change in expected output was less than 10 μV, smaller than the errors arising due to measurement and operational desynchronization.

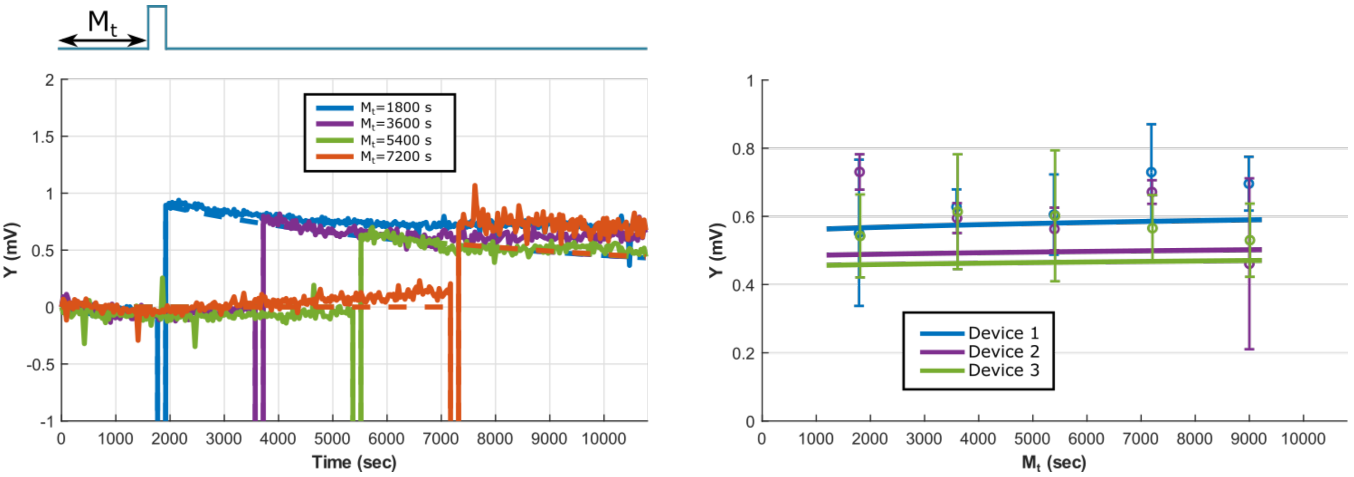

Supplementary Figure 11. Temporal dependence of recorder output to time of occurrence of an event. a) Recorder dynamics for events occurring at different times. b) Final output of three recorders, averaged over three trials, for events occurring at different times.

Supplementary References

[1] Lenzlinger, M. & Snow, E. Fowler-nordheim tunneling into thermally grown sio2. *Journal of Applied physics* **40**, 278–283 (1969).
